# Clinical phenotypes of motor neurone disease in a Kenyan hospital-based population

**DOI:** 10.3389/fneur.2025.1662690

**Published:** 2025-12-03

**Authors:** Effie Nailah Kamadi, Jasmit Shah, Juzar Hooker, Thomas M. Jenkins, Dilraj Singh Sokhi

**Affiliations:** 1 Department of Medicine, Aga Khan University Medical College of East Africa (Nairobi Campus), Nairobi, Kenya; 2 Brain and Mind Institute, Aga Khan University, Nairobi, Kenya; 3 Curtin University, Medical School (Perth Campus), Perth, WA, Australia

**Keywords:** motor neurone disease, amyotrophic lateral sclerosis, clinical phenotype, Sub-Saharan Africa/Kenya, hospital-based study, neurodegenerative disorders, HIV

## Abstract

**Background:**

Motor neurone disease (MND) presentation is globally heterogenous and data on the clinical phenotype in Sub-Saharan Africa (SSA) is scarce. We sought to address this by describing the profile of MND patients in a Kenyan hospital-based population.

**Methods:**

The medical charts of all adult MND patients assessed in the facility between January 2010 and December 2023 were retrospectively reviewed. The biographical data and clinical features of these patients were captured from their electronic and manual health records and statistical analysis performed.

**Results:**

In total, 160 patients had their data analyzed. The male to female ratio was 1.76:1. The median age at presentation was 55.0 (IQR: 45.0–68.0) years with a median diagnosis delay of 4.0 (IQR: 2.0–8.5) months. The site of first symptom onset was the lower limbs in 34.4% and the bulbar region in 33.1% [*95% CI (26.4–42.5%)*]. Notably, 59% of the patients were not tested for HIV and amongst those tested, 13.9% were HIV positive on ART. Majority (56.2%) of the patients were on Riluzole.

**Conclusion:**

This Kenyan case series of MND patients demonstrated a higher rate of bulbar onset disease [*33.1, 95% CI (26.4–42.5%), p = 0.018*] in comparison to what has been demonstrated in other African studies. A finding that supports geographic variation in MND presentation and that emphasizes the need for region specific genetic studies.

## Introduction

MND encompasses a rare group of neurological disorders characterized by relentless and progressive degeneration of upper and lower motor neurons leading to muscle weakness, disability and untimely death—often from respiratory failure (1, 2). Amyotrophic lateral sclerosis (ALS) is the most prevalent form of MND and it accounts for 80.0–90.0% of cases (3). Patient presentation and mode of disease onset is quite heterogeneous typified by limb and/or bulbar onset patterns and a median survival of 2–4 years (4).

Globally, peak disease onset ranges between 50.0 and 70.0 years with a male preponderance evidenced by a male to female ratio of ~1.3:1. In Western countries, between 60 and 75% of patients present with limb onset disease (4, 5). In Southern Germany, bulbar onset was noted in 34.1% of a cohort of MND patients (6) and in a Spanish population based observational study, the bulbar phenotype was found to be more prevalent amongst women and in subjects who were over 80.0 years old (7). These statistics on patient characteristics and clinical phenotype and progression of MND vary widely according to geography, race and ethnicity (8).

African MND has been demonstrated to show distinct epidemiological and clinical patterns that differ from what is observed in high income settings. Several studies—including the most comprehensive, multicentric study of ALS in Africa, the Tropical Amyotrophic Lateral Sclerosis (Tropals) study—have highlighted a younger age of onset ranging from 46.0 to 53.0 years, marked male preponderance with a male to female ratio of 2.9:1 and a predominance of spinal onset disease across North, West and South Africa. The bulbar onset phenotype comprised 22.7% of the patient cohort that was studied in Tropals and most of these cases were observed in Western Africa (9, 10). In Ethiopia, a multicenter hospital—based study revealed that 32.0% of patients exhibited bulbar onset (11).

A case series of 116 MND patients at a Tanzanian referral hospital suggested a higher prevalence of HIV infection of 17.0% amongst the cases of definite ALS compared to 4.7% in the general population (12, 22). Notably, Moodley et al. (13) found that HIV infected MND patients were younger at presentation with a median age of 41.0 years and had longer survival with slower disease progression when initiated on ART.

African MND literature is mostly from North, West and South Africa with an evident paucity in studies in comparison to the rest of the globe (14). In East Africa, the published studies are from Tanzania (2018) and Kenya (1992) and they were limited by a lack of diagnostic electromyography (EMG) studies and missing data (22). There is need to describe the phenotype of MND in this understudied population as this provides current local data and an updated understanding of the evolving regional clinical patterns. This will provide much needed background for multicentric genetic research. Anecdotal evidence from the AKUHN neurology section revealed a much higher proportion of bulbar onset cases, which contradicted the findings of other African investigations. We sought to explore this statistically by investigating the clinical phenotype and outcomes of Kenyan MND patients seen at a tertiary, private funded referral facility between January 2010 and December 2023.

## Study objectives

### Primary objective

To describe the clinical phenotype of MND amongst adults presenting at the AKUHN neurology department.

### Secondary objective

To determine the outcomes of MND patients presenting at the AKUHN neurology department.

*Note:* Due to high rates of loss to follow up, outcome data was not consistently recorded and was therefore not analyzed. As such, this objective was not fulfilled.

## Materials and methods

### Study site

This study was performed at the AKUHN which is a tertiary, private – funded, referral facility located in Nairobi, Kenya. It serves the East and Central African population.

### Study design

This was a retrospective descriptive chart review done on file records from January 1st, 2010, to December 30th, 2023. The study involved collecting information from files within the medical records department. All available charts with MND between January 2010 and December 2023 were reviewed. Case finding was done using the following methods:

An ICD-10 code (G12.20) search in medical records offices.

Search for EMG studies that diagnosed MND.

Search in the medical pharmacy was done to find patients who have had prescriptions for drugs used in MND especially Riluzole.

Patient records under a private neurologist were obtained using proxy information.

### Study population

The study population was all patients aged above 18.0 years evaluated in AKUHN and diagnosed with MND.

#### Inclusion criteria

Age >18.0 years at the time of diagnosis.
Medical records of patients with clinical and electrophysiological diagnosis of MND that fulfil the Gold Coast criteria within the study duration at AKUHN.
Repetition of clinical examinations at least 6.0 months apart to ascertain evidence of progression.

#### Exclusion criteria

Medical records of patients with electrophysiological or neuroimaging evidence of other disease processes that might explain the signs of motor degeneration.

### Sample size

All patients diagnosed with MND based on EMG studies within the study duration (January 2017 to December 2023) were included. A total of one hundred and sixty patient records were reviewed.

### Data management

All patient files with an MND diagnosis were identified and extracted from the AKUHN medical records and reviewed. Electrophysiological diagnostic reports (i.e., EMG reports that are diagnostic of MND) were also obtained from the neurology clinic. The electronic document management system provided further information when needed. Data was collected on patient characteristics, their clinical features and neurological examination findings at review in the neurology clinics as well as their use of Riluzole. This was based on the data abstraction tool as attached in the Appendix.

Data was recorded using Research Electronic Data Capture (REDCap) which was only accessible to the principal investigator and the supervisors. Patients with missing data with the variables of interest were excluded from the data analysis. The patients’ hospital identification numbers were removed, and patients were assigned a study number. In the event a patient had 2 Aga Khan (AK) numbers, only one was recorded to avoid double entry. Study ID numbers were linked to AK numbers in a separate Excel Sheet that was password—protected and stored on the Aga Khan University Microsoft OneDrive folder of the principal investigator, which was only accessible with two-step authentication. The Statistical Package for the Social Sciences (SPSS) was used for data analysis. At the end of the study, all data was handed over to the Research Office at the Aga Khan University for management as per institutional policy.

#### Data analysis

Continuous data were presented as means with standard deviations or as medians with interquartile ranges, whilst categorical data were presented as frequencies and percentages with corresponding confidence intervals. Analyses were primarily descriptive in nature and were conducted on available data per variable. No multivariable testing was performed due to the limited sample size and extent of missing data. A *p*-value of <0.05 was considered statistically significant for any comparisons performed.

### Ethical considerations

Ethical approval was obtained from the Institutional Scientific and Ethics Review Committee, ISERC *(Ref: 2023/ISERC-81 (v1))* at the Aga Khan University as well as from the National Commissions for Science, Technology and Innovation *(License No: NACOSTI/P/23/29928)*. Since this was a retrospective descriptive study and did not entail human participation, a waiver of consent was secured. Patient privacy was protected as all data was de-identified for analysis.

## Results

### Patient characteristics

Data was collected for 160 MND patients. Of these, 34.4% (*n* = 55) only had a diagnostic EMG performed after being referred from other facilities, whereas 65.6% came in for neurologic evaluation of their symptoms. The male to female ratio was 1.76:1 with males being 102 (63.8%) in number. The observed median age was 55.0 (IQR: 45.0–68.0) years. The youngest age at which a diagnosis of MND was made was 27.0 years and young onset disease (<30.0 years) was observed in 5 (3.1%) of the 160 patients.

### Clinical features

There were missing data on 9 patient records on the site of first symptom onset and these were therefore excluded on the analysis of this clinical feature. Final analysis of the remaining 151 patient records revealed that 34.4% had lower limb onset, 33.1% had bulbar onset, 31.8% had mixed presentation, 22.5% had upper limb onset and 8.6% had respiratory onset. Amongst the cases that had bulbar onset, 50.0% were female.

Of the 151 patients with data on the site of first symptom onset, 46 had no clinical feature data available. Further analysis was therefore conducted on the remaining 105 patients (151 − 46 = 105). More data on clinical features was unavailable for 46 patients as these had only been referred to AKUHN by their primary care providers with a brief clinical history for EMG studies that eventually confirmed an MND diagnosis. Therefore, further clinical features were only recorded and analyzed for the remaining 105 patients who were evaluated in the neurology clinics. Since the date of first symptom onset, the median duration of time before these patients were seen and diagnosed (diagnostic delay) was 4.0 (IQR: 2.0–9.0) months. Notably, the date of first muscle weakness was not recorded for 5 of the patients and the duration of time before seeing a doctor was not recorded for 8 patients. Data on diagnostic delays was therefore analyzed from 92 patients.

At the first neurological assessment, most (55.2%) of the patients presented with symptoms of leg weakness, 43.8% presented with arm weakness, 42.9% presented with bulbar weakness, 14.3% presented with respiratory weakness, 10.5% presented with foot drop, 6.7% presented with cognitive decline, 5.7% presented with muscle cramps and 1.0% presented with head drop.

Amongst the 105 patients, 59% (62) were not tested for HIV and of the 43 patients who were screened, 13.9% (6) were HIV positive on Anti-Retroviral Therapy. A small minority (1.9%) reported familial MND.

On neurological examination, 47.6% had leg LMN signs, 35.2% had arm LMN signs, 31.4% had bulbar UMN signs, 26.7% had bulbar LMN signs, 27.6% had leg UMN signs and 26.7% had arm UMN signs. Based on this neurological examination findings clinical phenotype was assigned and 58.1% had ALS, 21.0% had PMA and 21.0% had PLS.

### Patient evaluation and management

As part of the patients’ work up, laboratory and imaging studies were conducted to rule out any conditions that would explain the symptoms. Data on these investigations were incomplete and hence not included in statistical analysis.

Once a diagnosis of MND was arrived at, 56.2% of patients were noted to have been on Riluzole for treatment. For swallow assessment, 27.0% of the patients were reviewed by the speech and language therapist and 11.4% had a PEG tube placed. Assessment for type II respiratory failure with FVC measurement was only performed on 8.6% of patients. Lastly, only 18.1% were referred to palliative care.

Data on revised El Escorial criteria, ALS—Functional Rating Scale (ALS—FRS) scores for assessment of functionality at first and last review, presence of advance directives, date of demise and cause of death were unavailable, and these were therefore not analyzed.

## Discussion

### Patient characteristics

**Figure 1:**
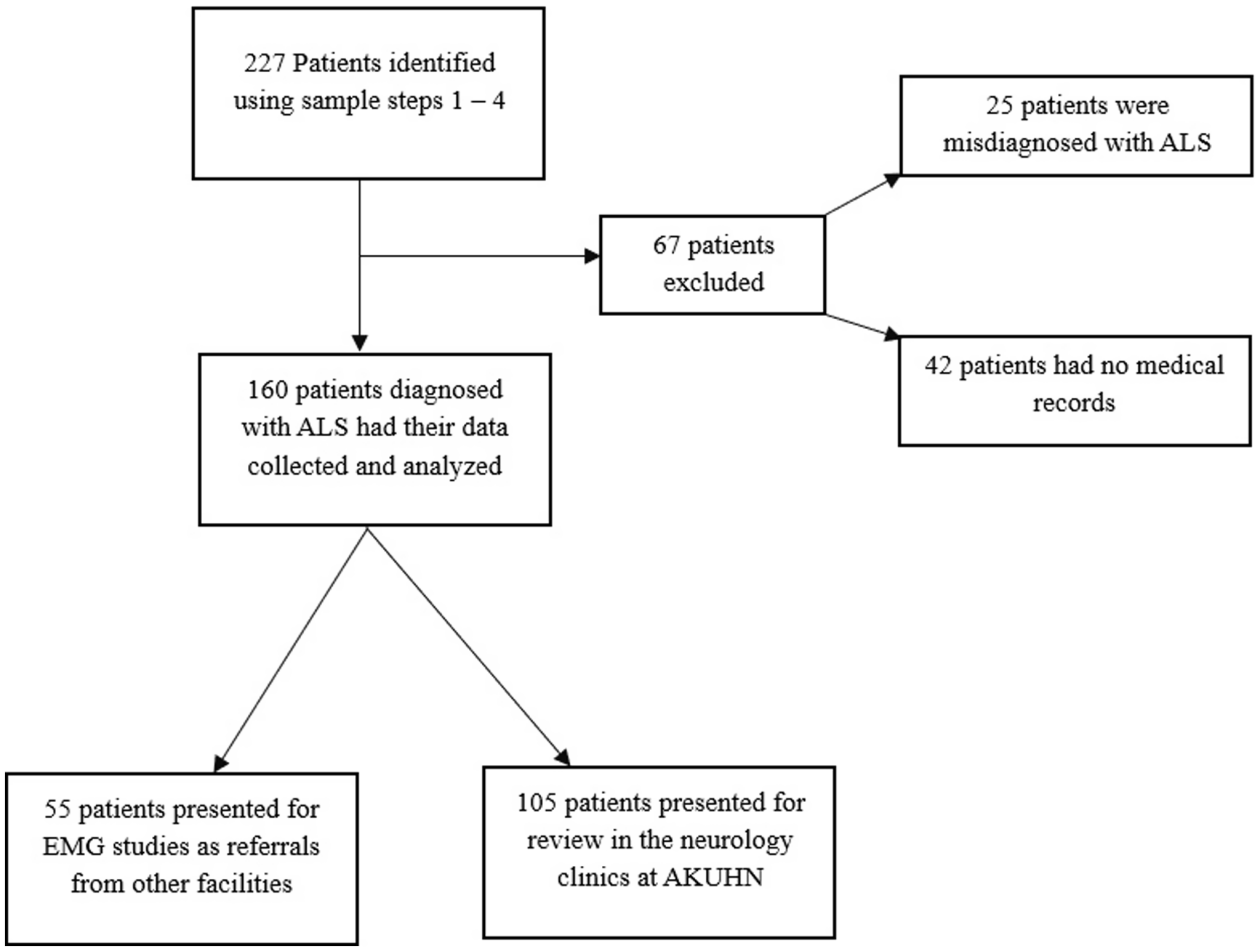
Summary of the data collection procedure.

This retrospective analysis constitutes one of the largest MND case series in SSA (*n* = 160) and it entails a characterization of the clinical features of a rare, understudied, debilitating and fatal neurological ailment. Notably, the condition’s presentation in our setting is largely similar to that in other countries, however, we demonstrate certain significant differences in the Kenyan phenotype. The patient selection and data collection process are summarized in Figure 1 detailing the number of records screened, excluded and included in the final analysis (Table 1).

**Table 1:** Demographics & Patients’ Characteristics

**Type of source (N = 160)**	Medical records	105	65.6%
	No files (referrals from other institutions)	55	34.4%
**Gender** **(N = 160)**	Males	102	63.8%
	Females	58	36.2%
**Age in years, [IQR] (N = 160)**		55.0 [45.0, 68.0]	
**Diagnostic delay in months, [IQR] (n = 92)** ^∆^		4.0 [2.0, 9.0]	
**Residence (n = 103)** ^∆^	Rural	9	8.7%
	Urban	94	91.3%
**Marital status (n = 98)** ^∆^	Married	77	78.6%
	Single	21	21.4%
**Site of first symptom onset (n = 151)** *	Lower limb	52	34.4%
	Bulbar	50	33.1%
	Mixed presentation	48	31.8%
	Upper limb	34	22.5%
	Respiratory	13	8.6%
**Clinical features at first neurological assessment (n= 105)** *	Leg weakness	58	55.8%
	Arm weakness	46	44.2%
	Bulbar weakness	45	43.3%
	Respiratory weakness	15	14.4%
	Foot drop	11	10.6%
	Cognitive decline	7	6.7%
	Cramps	6	5.8%
	Head drop	1	1.0%
**Familial MND (N = 105)**		2	1.9%
**HIV testing status (N = 105)**	Tested	43	41.0%
	Not tested	62	59.0%
**Among tested (n = 43)**	HIV positive (on art)	6	13.9%
	HIV negative	37	86.1%
**Neurological examination (N = 105)** *	Bulbar UMN signs	33	32.4%
	Bulbar LMN signs	28	27.5%
	Arm UMN signs	28	27.5%
	Arm LMN signs	37	36.3%
	Leg UMN signs	29	28.4%
	Leg LM signs	50	49.0%
**Clinical phenotype (N = 105)**	ALS	61	58.1%
	PMA	22	21.0%
	PLS	22	21.0%
**Riluzole use (N = 105)**	59		56.2%
**Swallow assessment (N = 100)** ^∆^	Reviewed by speech and language therapist (SLT)	27	27.0%
	Never reviewed by SLT	73	73.0%
**PEG in situ (N = 105)**		12	11.4%
**FVC done (N = 105)**	Yes	9	8.6%
	No	96	91.4%
**Palliative care referral (N = 105)**	Yes	19	18.1%
	No	85	80.1%

*Percentages exceed 100% because multiple clinical features could be present in a single patient.

∆Denominators vary due to missing data on specific variables.

#### Age

The median age at presentation of all the patients diagnosed with MND was 55.0 (IQR: 45.0–68.0) years. We noted 5 (3.1%) cases had MND diagnosed before 30 years of age with the youngest patients being 27.0 years. Globally, the median age of MND disease onset is in the seventh decade (15) and it is has been demonstrated that compared to African Americans, Caucasians were significantly older at diagnosis (*61.0 vs 55.0 years, p = 0.011*) (16). Our finding of a much earlier age of onset of 55.0 years corroborates this fact.

Similarly, in the TROPALS study, disease onset was noted to happen at a median age of 53.0 years with West African patients being significantly younger (47.0 years) than those in North and South Africa (*p = 0.0003*). The proportion of young onset MND cases was 4.3% (10). In Ethiopia, the mean age of disease onset was found to be 51.9% with 2.9% of patients presenting with juvenile MND (11). A Tanzanian case series found an average age of onset at 53.7 (IQR: 15.0–91.0) years.

It follows that our finding of an earlier age of onset (55.0 years) with a low proportion of young onset ALS (3.1%) is in keeping with findings of studies done across Africa. The reasons for this younger age of onset observed amongst African patients is not exactly elucidated however there are proposed theories that implicate the higher prevalence of HIV amongst Africans as a predisposition to early onset motor neuron syndromes (13). Firm conclusions about this association, however, cannot be made and further studies are needed. Genetic, environmental, or healthcare access factors may also underpin this difference in age onset across racial and ethnic lines. Additionally, the difference in population structure of Western and African regions may contribute to the age difference rather than intrinsic differences in disease manifestation (10).

#### Gender differences

The male to female ratio of 1.76:1 amongst all patients diagnosed with MND at AKUHN is comparable to the gender ratio of 2.9:1 reported in the TROPALS study that included data from multiple African studies (10). A chart review of 71 MND patients who attended clinic at an academic hospital in Soweto, South Africa as well as an Ethiopian multicenter retrospective study found a male to female ratio of 2:1 (9, 11). A case series of 116 patients in a Tanzanian referral hospital reported a ratio of 1.63:1.

Consequently, our gender ratio is comparable to what is known in Africa as well as globally where the ratio is ~1.3 (4). We also note that our gender ratio is slightly higher than what is observed in the West; a finding that was also suggested by multicenter cohort studies of MND in Africa (9, 11). This slightly higher male preponderance in Africans could be because of a combination of genetic, hormonal, physiological or environmental factors. Misdiagnosis or decreased exposure to environmental or occupational risk factors amongst female patients may explain this finding (17). It may also reflect a referral bias with disparities in healthcare access and diagnosis across the genders.

#### Residence

Most (91.3%) of our patients were of urban residence, likely reflecting the tertiary referral nature of our center and potential under – representation of rural populations. While a small case—control (23 ALS cases, 53 controls) study in Senegal identified living outside Dakar [*OR: 16.4; 95% CI (2.6–69.9)*] and pesticide exposure [*OR: 15.3; 95% CI (3.7–63.4)*] as main risk factors for ALS (18), we report a case series of MND patients, the majority of whom dwelled in urban centers. This discrepancy may suggest better case identification in urban centers due to better access to health care with underdiagnosis in rural areas. Alternatively, there may be distinct environmental risk factors such as air pollution, industrial exposures or dietary patterns unique to the urban setting that may contribute to MND risk. This warrants further investigation by way of large multi – center or population-based studies. Understanding this nuance is pertinent for refining risk factor assessments in diverse settings.

### Clinical features

Sub-Saharan Africa is thought to exhibit distinct patterns of neurological disease but with MND subtypes that are consistent with what is described globally (19, 20).

#### Site of first symptom onset

**Figure 2:**
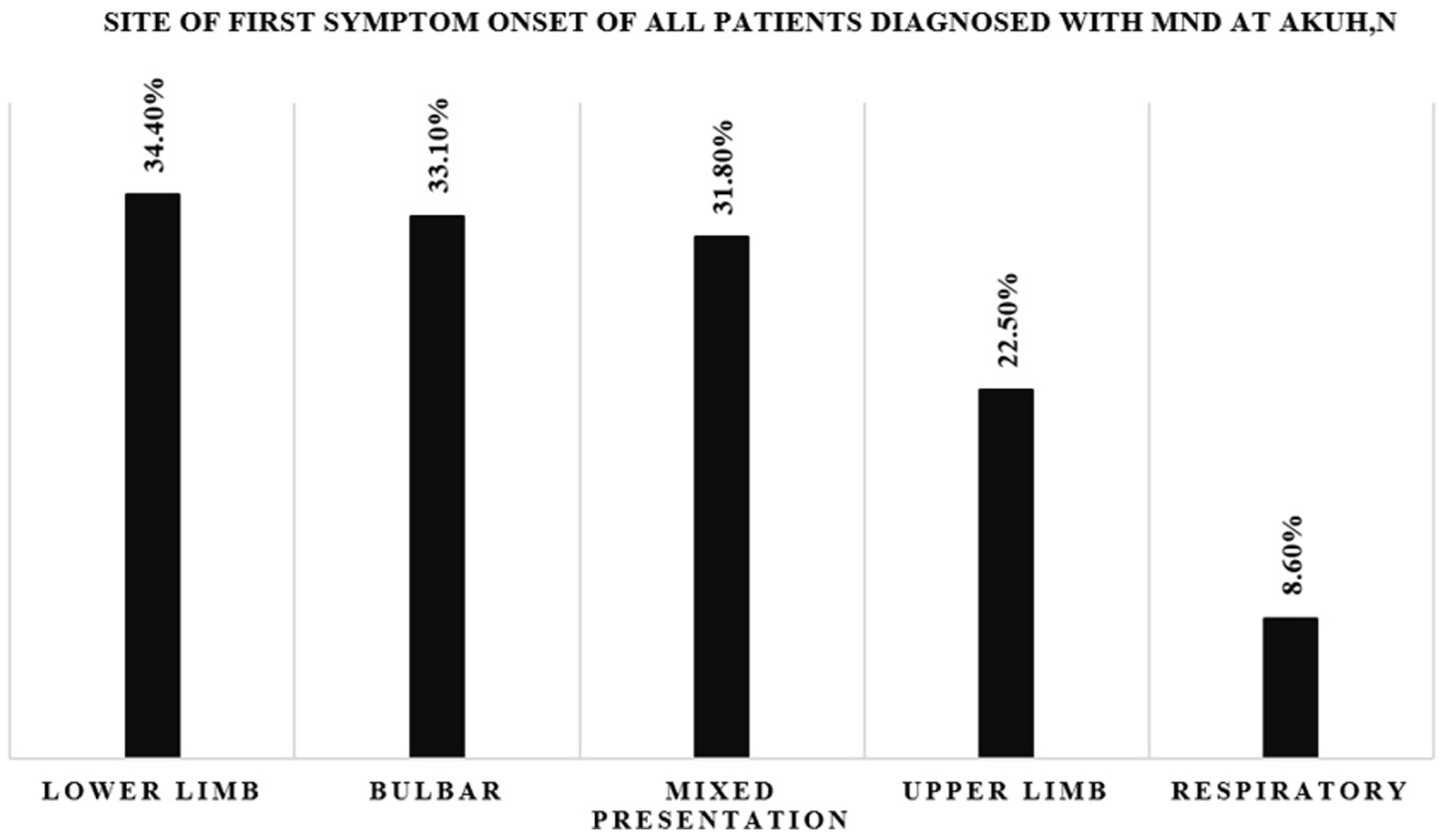
Bar graph showing the site of first symptom onset for all patients diagnosed with MND at AKUHN.

Analysis of all the patients (151) who had complete records on the mode of disease onset (see Figure 2) revealed that 34.4% had lower limb at onset and 33.1% [*95% CI (26.4–42.5%)*] had bulbar onset. Notably, 50% of these patients with bulbar onset were female. The least common phenotype was respiratory onset MND (8.6%).

In the West, the proportion of bulbar onset MND is heterogeneous with 34.1% of patients in a German study presenting in this manner (6). In a Spanish study, this bulbar phenotype was mostly seen in women and in much older patients above 80.0 years (7). The TROPALS study documented a much lower percentage [*22.7, 95% CI (17.2–29.3%), p = 0.018*] of bulbar onset disease with most cases being from West Africa (10). A series of 71 South African MND patients showed a bulbar onset proportion of 23% [*95% CI (13.0–36.0%)*] had bulbar onset (9).

In this study, the proportion of bulbar onset MND of 33.1% [*95% CI (26.4–42.5%)*] is significantly higher compared to what was found in the TROPALS study [*22.7, 95% CI (17.3–29.3%), p = 0.018*]. Our proportion of bulbar onset MND align closely with that from an Ethiopian multicenter retrospective analysis [*32, 95% CI (22.0–43.0%)*] with a similar confidence range suggesting a unique MND phenotype in Eastern Africa. We also noted a male: female ratio amongst these bulbar onset cases of 1:1 which contradicts the finding of female preponderance amongst bulbar onset cases in a Spanish study (7).

Possible explanations for these include unique genetic factors amongst East Africans as well as dietary or environmental differences. It is worth noting that majority of cases in our study had lower limb onset disease. The observed proportion of bulbar onset MND may reflect faster clinical progression and earlier healthcare-seeking in these patients (6). In contrast, limb onset MND may progress insidiously and remain underdiagnosed in the early stages.

#### Diagnostic delay

Based on the available diagnostic timeline data from 92 patients, a 4.0 (IQR 2.0–9.0) month diagnostic delay was seen, which is significantly less than what has been reported in other studies. The TROPALS study reported a median diagnosis delay of 10.5 months (6.0–20.5 months) (10), while a South African case series documented a longer delay of 2.0 (IQR: 1.0–3.0) years (9). This shorter diagnostic delay may be attributed to the higher prevalence of bulbar onset MND, which results in more incapacitating symptoms such as speech and swallow difficulty, prompting patients to seek earlier medical attention. It may also indicate increased awareness of neurology services in Kenya and more efficient referral pathways for diagnostic testing. A lower prevalence of conditions that mimic MND symptoms in Kenya may also prompt patients to seek care faster, leading to an early diagnosis of MND. There is also likely to be a referral bias as these patients were seen at a tertiary center having been referred for specialist review.

#### Comorbid HIV

Amongst the 105 patients reviewed in the neurology clinics, HIV status was not assessed in 62 (59%). Of those tested, 6 (13.9%) were HIV positive on ART. Given that HIV is highly endemic in the SSA and it has been shown to be linked to motor neuron syndromes, this degree of undertesting is concerning. Routine HIV screening in patients with suspected or confirmed MND is important, particularly since HIV associated motor neuron syndromes have shown clinical improvement with early ART initiation (13).

Potential contributors to low testing rates include assumptions of low risk in patients without the traditional risk factors for HIV, the diagnostic overshadowing by neurological symptoms and patient reluctance to consent to the test due to stigma.

#### Riluzole use

In this study, 56.2% of the MND patients were on Riluzole therapy. This is notably higher than the 26.3% reported in the TROPALS study (10) and the 31.0% observed in a recent Ethiopian multicenter retrospective review (11). Riluzole confers a modest survival benefit in MND, yet its access remains limited largely due to its prohibitive price (21). The relatively higher uptake (56.2%) in our case series may connote better access to neurology services given that most 91.3% of these patients were of urban residence. However, there is still a need to improve equitable access to Riluzole amongst patients.

### Study limitations

Limitations of this study included irregular records of data on the El Escorial criteria, EMG results and ALS-FRS scores for the included patients and therefore these were excluded from data analysis. Since most patients were lost to follow-up, records of patient deaths and the presence of advance directives before this were missing and therefore analysis of the outcomes of MND patients seen at our clinic was not possible. This is likely because majority of patients may have passed away at home or in nearby hospice facilities. As a result, we failed to fulfil the secondary objective of our study, which was to describe the outcomes of MND patients.

## Conclusion

This study addresses the knowledge gap on the characteristics and clinical phenotype of MND patients in East Africa. It included one of the largest case series of MND patients (*n* = 160) in a hospital-based population. We found an earlier median age of onset (55.0 years) with 3.1% of the patients presenting with young onset MND. This earlier age of disease onset was consistent with what is known about African MND. We also noted a slightly higher gender ratio in comparison to Western literature (1.76:1 vs. ~ 1.3:1); an observation that has also been noted in existing African MND literature.

The majority (34.4%) of cases in our study had lower limb onset disease and the least common phenotype was respiratory onset MND (8.6%). A statistically significant difference was noted in the proportion of bulbar onset MND in our study [*33.1, 95% CI (26.4–42.5%), p = 0.018*] in comparison to what is reported in the rest of the African subcontinent. This adds to the growing body of evidence supporting geographical variation in disease presentation. There is a need for further genetic studies to explore the underlying mechanisms for this heterogeneity. This may ultimately inform the development of targeted treatment strategies. We noted that HIV testing was underperformed as 59% of the patients were untested and that 13.9% of the 43 tested patients were HIV positive on ART. Slightly over 50% of the patients were on Riluzole which was a higher proportion compared to what was found in other African studies and which reflects a persisting disparity in access to guideline based MND treatment.

## Data Availability

The raw data supporting the conclusions of this article will be made available by the authors, within reasonable request.
