# Supplementary material for: Clinical phenotypes of motor neurone disease in a Kenyan hospital-based population
Source: Front Neurol. 2025 Dec 3;16:1662690. doi: 10.3389/fneur.2025.1662690 (PMC12710236; doi:10.3389/fneur.2025.1662690)
Supplement: Supplementary file 1 [file Data_Sheet_1.pdf]

DEMOGRAPHICS & PATIENTS' CHARACTERISTICS

**Type Of Source (N = 160)**	Medical records	105	65.6%
	No files (referrals from other institutions)	55	34.4%
**Gender (N = 160)**	Males	102	63.8%
	Females	58	36.2%
**Age In Years, [IQR] (N = 160)**		55.0 [45.0, 68.0]	
**Diagnostic Delay in months, [IQR] (n = 92)**		4.0 [2.0,9.0]	
**Residence (n = 103)**	Rural	9	8.7%
	Urban	94	91.3%
**Marital Status (n = 98)**	Married	77	79.4%
	Single	21	21.6%
**Site Of First Symptom Onset (n = 151)***	Lower limb	52	35.4%
	Bulbar	50	34.0%
	Mixed presentation	48	32.7%
	Upper limb	34	23.1%
	Respiratory	13	8.8%
**Clinical Features at First Neurological Assessment (n = 105)***	Leg weakness	58	55.8%
	Arm weakness	46	44.2%
	Bulbar weakness	45	43.3%
	Respiratory weakness	15	14.4%
	Foot drop	11	10.6%
	Cognitive decline	7	6.7%
	Cramps	6	5.8%
	Head drop	1	1.0%
**Familial MND**		2	1.9%
**HIV Testing Status (N = 105)**	Tested	43	
	Not tested	62	41.0%
**Among Tested (n = 43)**	HIV positive (on art)	6	13.9%
	HIV negative	37	86.1%
**Neurological Examination (N = 105)***	Bulbar UMN signs	33	32.4%
	Bulbar LMN signs	28	27.5%
	Arm UMN signs	28	27.5%
	Arm LMN signs	37	36.3%
	Leg UMN signs	29	28.4%
	Leg LM signs	50	49.0%
**Clinical Phenotype (N = 105)**	ALS	61	59.8%
	PMA	22	21.6%
	PLS	22	21.6%
**Riluzole Use (N = 105)**	59		56.7%
**Swallow Assessment (N = 105)**	Reviewed by speech and language therapist (SLT)	27	26.0%
	Never reviewed by SLT	73	70.2%
	Peg in situ	12	11.5%
**FVC Done (n = 102)**	Yes	9	8.8%
	No	96	94.1%
**Palliative Care Referral (N = 105)**	Yes	19	18.3%
	No	85	81.7%

**Percentages exceed 100% because multiple clinical features could be present in a single patient.*
